# Comparison of biological characteristics of mesenchymal stem cells derived from maternal-origin placenta and Wharton’s jelly

**DOI:** 10.1186/s13287-015-0219-6

**Published:** 2015-11-25

**Authors:** Gecai Chen, Aihuan Yue, Zhongbao Ruan, Yigang Yin, Ruzhu Wang, Yin Ren, Li Zhu

**Affiliations:** Department of Cardiology, Taizhou Renmin Hospital, Taizhou, Jiangsu Province China; Stem Cell Research Center, Taizhou, Jiangsu Province China

**Keywords:** Mesenchymal stem cells (MSCs), Decidua basalis, Wharton’s jelly, Immunosuppression, Cell cycle, T-cell proliferation

## Abstract

**Introduction:**

Although mesenchymal stem cells (MSCs) from different sources share many similar characteristics, they also exhibit individual properties. In this study, we compared MSCs derived from Wharton’s jelly in the umbilical cord with those derived from the decidual basalis in the maternal part of the placenta to better understand the similarities and differences between these two cell types.

**Method:**

The morphology, immunophenotype (as assessed using flow cytometry), and multi-lineage differentiation potential were analyzed. Karyotype analysis was carried out to determine the origin of the MSCs. Growth kinetics were evaluated using analysis of the population doubling time and cell cycle. Immunosuppressive function was analyzed using mixed lymphocyte culture.

**Results:**

MSCs from Wharton’s jelly and the decidua basalis exhibited similar morphology, immunophenotype, and differentiation potential to osteogenesis and adipogenesis. The percentage of MSCs in the G0/G1 phase was higher in the case of Wharton’s jelly than in the case of the decidua basalis (*P <* 0.05). Decidual MSCs displayed more remarkable immunosuppressive effects on phytohemagglutinin-stimulated T-cell proliferation (*P <* 0.05).

**Conclusion:**

MSCs from both sources had similar basic biological properties, but decidual MSCs had slower proliferation and stronger immunosuppressive function.

## Introduction

Mesenchymal stem cells (MSCs) not only possess the basic characteristics of stem cells, including self-renewal and multi-lineage differentiation potential, but also exhibit hematopoietic [1, 2] and immunomodulatory function [3–6]. Neonatal tissue is rich in MSCs derived from Wharton’s jelly in the umbilical cord and from the deciduae, which form the maternal part of the placenta. The placentome is customarily discarded as a medical waste, and there is no ethical controversy in obtaining MSCs from this tissue. There may be many similarities between MSCs from the above two sources. Nevertheless, they play different roles during fetal development, and so have their own characteristics. The placenta and fetal membranes function as immunological barriers between the mother and the developing fetus during pregnancy. The placenta can be conceptually divided into the fetal side, consisting of the amnion and chorion, and the maternal side, consisting of the decidua. As placental tissues are conventionally discarded after delivery, these tissues are readily available for research and clinical applications. The decidua is a membrane of maternal origin that plays an important role in immune tolerance, since maternal and fetal immune cells come into direct contact with each other at this site [5]. Wharton’s jelly is the embryonic mucous connective tissue found between the amniotic epithelium and the umbilical vessels; it is a rich source of MSCs [7]. MSCs from Wharton’s jelly (WJ-MSCs) exhibit greater proliferation than adult MSCs from the bone marrow [6].

Most often MSCs are transplanted for tissue repair and regeneration. Due to their immunomodulatory properties, MSCs have garnered increasing research attention in recent years. MSCs have been used for treating graft-versus-host disease [5, 8–10]. MSCs from the bone marrow, which were first described by Fridenstein et al. [11] in 1976, were the earliest stem cells to be detected and, currently, are the most used stem cells in clinical trials. However, their limited availability hindered their development in research and clinical applications. The use of neonatal tissue can overcome this shortcoming. In our study, we compared MSCs derived from Wharton’s jelly in the umbilical cord and from the decidual stroma in the maternal-origin placenta to understand their similarities and differences. The morphology and immunophenotype (assessed using flow cytometry) were analyzed. Karyotype analysis was carried out to determine the origin of the MSCs. Growth kinetics were evaluated using the population doubling time (PDT) and cell cycle. Immunosuppressive function was analyzed using mixed lymphocyte culture.

## Materials and methods

### Isolation and culture of MSCs from Wharton’s jelly and decidua

Ten human placentae and umbilical cords were obtained from healthy, full-term, naturally delivered, male newborns. Peripheral blood samples were obtained from voluntary blood donors. Written informed consent was obtained from the mothers and the donors. The study protocols were reviewed and approved by the Taizhou Renmin Hospital review board and ethics committee of Taizhou Renmin Hospital. We selected donors who tested negative for hepatitis B surface antigen, hepatitis B core antibody, hepatitis C virus antibody, hepatitis C virus RNA, HIV-I and -II antibodies, HIV-1 RNA, cytomegalovirus IgM, and anti-*Treponema pallidum* antibody.

WJ-MSCs were separated and cultured according to previously published reports [11, 12]. MSCs from the decidua basalis (DB-MSCs) were separated from the decidua basalis of the placenta. The decidua basalis tissue was sliced into small fragments of 1 mm^3^, washed twice with physiological saline, digested with collagenase for 1 h, and cultured in serum-free MesenCult-XF medium (Stemcell, Vancouver, Canada).

### Karyotype analysis

Karyotype analysis was carried out at passage 0 (P_0_) to confirm that the cells were derived from the maternal decidua basalis. For this purpose, 2 × 10^6^ cells were harvested, and 0.1–0.4 μg/mL colchicine (Gibco, Grand Island, USA) was added to the culture medium. After 12 h, 0.075 M KCl was added to the culture, and the cells were incubated in a water bath at 37 °C. Then, 1 mL of fixative (methanol/acetic acid mixture at 1:3) was added, and the samples were incubated for 30 min at 37 °C and centrifuged. A further 8 mL of fixative was added, and the cells were dried for 10 min with 10 % Giemsa, and then washed with distilled water. The fixed cells were observed under an electron microscope (IX71; Olympus, Tokyo, Japan). Chromosome analysis was carried out by applying G-bands, according to the guidelines of the International System for Chromosome Nomenclature 2013. On average, 20 metaphase samples were evaluated for each passage [13].

### Immunophenotype analysis by flow cytometry

At P_3_, MSCs from both sources (1 × 10^7^ cells) were digested with trypsin and washed twice with phosphate-buffered saline. The cell concentration was adjusted to 2 × 10^6^ cells/mL, and cells were stained with the following fluorescent antibody conjugates: CD45-fluorescein isothiocyanate (FITC), CD34-phycoerythrin (PE), CD73-PE, CD14-FITC, CD79a-APC, the human major histocompatibility complex (MHC) class II molecule HLA-DR-(PE), CD90-allophycocyanin (APC) (BD Biosciences, MD, USA), and CD105-PE (eBioscience, CA, USA). We also tested for the co-inhibitory molecule B7-H1(FITC) and the positive co-stimulatory factors CD80-PE, CD83-APC, and CD86-FITC. Surface staining was detected using flow cytometry (Diva software 6.0, FACScantoII, BD Biosciences).

### Growth kinetics analysis

The proliferation of MSCs from both sources at P_3,_ P_5,_ P_8_, and P_10_ was assessed. WJ-MSCs and DB-MSCs were plated on a 60-mm wide dish at a density of 7–10 × 10^5^ cells/well, and the cells were counted until they reached 100 % confluency. The PDT was calculated using the following formula:

PDT = (CT × ln2)/ln(N_f_/N_i_), where CT is the cell culture time, N_i_ is the initial number of cells, and N_f_ is the final number of cells [14].

### Cell cycle analysis of MSCs from both sources by flow cytometry

Cell cycle analysis was carried out at P_3_. The cell concentration was adjusted to 2 × 10^6^ cells/mL. A 1-mL cell suspension in 70 % ethanol containing 1 × 10^6^ cells was prepared and fixed for 10–12 h at 4 °C. The fixed cells were centrifuged for 5 min at 300 *g*. The supernatant was discarded, and the cells were stained with 1 μg/mL propidium iodide (BD Biosciences). The cells were incubated for 20 min at 4 °C, and their fluorescence was measured using flow cytometry. The data were analyzed using ModFit software.

### Mixed lymphocyte reaction

We gently mixed 10 mL peripheral blood with 10 mL saline. Next, 10 mL lymphocyte separation medium 1.077 was poured into a 5.0-mL tube. Then, 20 mL cell suspension was carefully added on top of the separation medium without disturbing the interphase. The tube was centrifuged at 440 *g* for 40 min. Most of the supernatant was then aspirated without disturbing the layer of mononuclear cells in the interphase. The mononuclear cells were then aspirated from the interphase, washed with saline, and centrifuged at 360 *g* for 10 min. The excess red blood cells and plasma were removed.

Mixed lymphocyte reaction was carried out in 96-well plates. WJ-MSCs and DB-MSCs from 10 donors at P_3_ were irradiated with ^60^Co (20 Gy). Next, 1.0 × 10^5^ responder cells were co-cultured with 1.0 × 10^5^ stimulator cells in serum-free MesenCult-XF medium for 6 days at 37 °C in humidified air containing 5 % CO_2_. The cells were divided into eight groups: group A, 1.0 × 10^6^ peripheral blood mononuclear cells (PBMCs); group B, 1.0 × 10^6^ PBMCs + phytohemagglutinin (PHA; 10 ug/mL); group C, 1.0 × 10^5^ DB-MSCs; group D, 1.0 × 10^5^ DB-MSCs + PHA; group E, 1.0 × 10^6^ PBMCs + 1.0 × 10^5^ DB-MSCs + PHA (10 μg/mL); group F, 1.0 × 10^5^ WJ-MSCs; group G, 1.0 × 10^5^ WJ-MSCs + PHA; group H, 1.0 × 10^6^ PBMCs + 1.0 × 10^5^ WJ-MSCs + PHA. For each group, three replications were used. Cell proliferation rates were assessed using (^3^H)-thymidine incorporation. The interferon (IFN)-γ levels in the co-culture supernatant were detected using an enzyme-linked immunosorbent assay (ELISA) kit (eBioscience). The optical density of each well was evaluated at 450/630 nm, and IFN-γ content was calculated using a standard curve.

### Statistical analysis

Data were expressed as mean ± SEM. The different groups were compared using analysis of variance. PDT was compared using the *t*-test. A 5 % probability (*P* < 0.05) was used as the level of statistical difference.

## Results

### Morphology

**Fig. 1:**
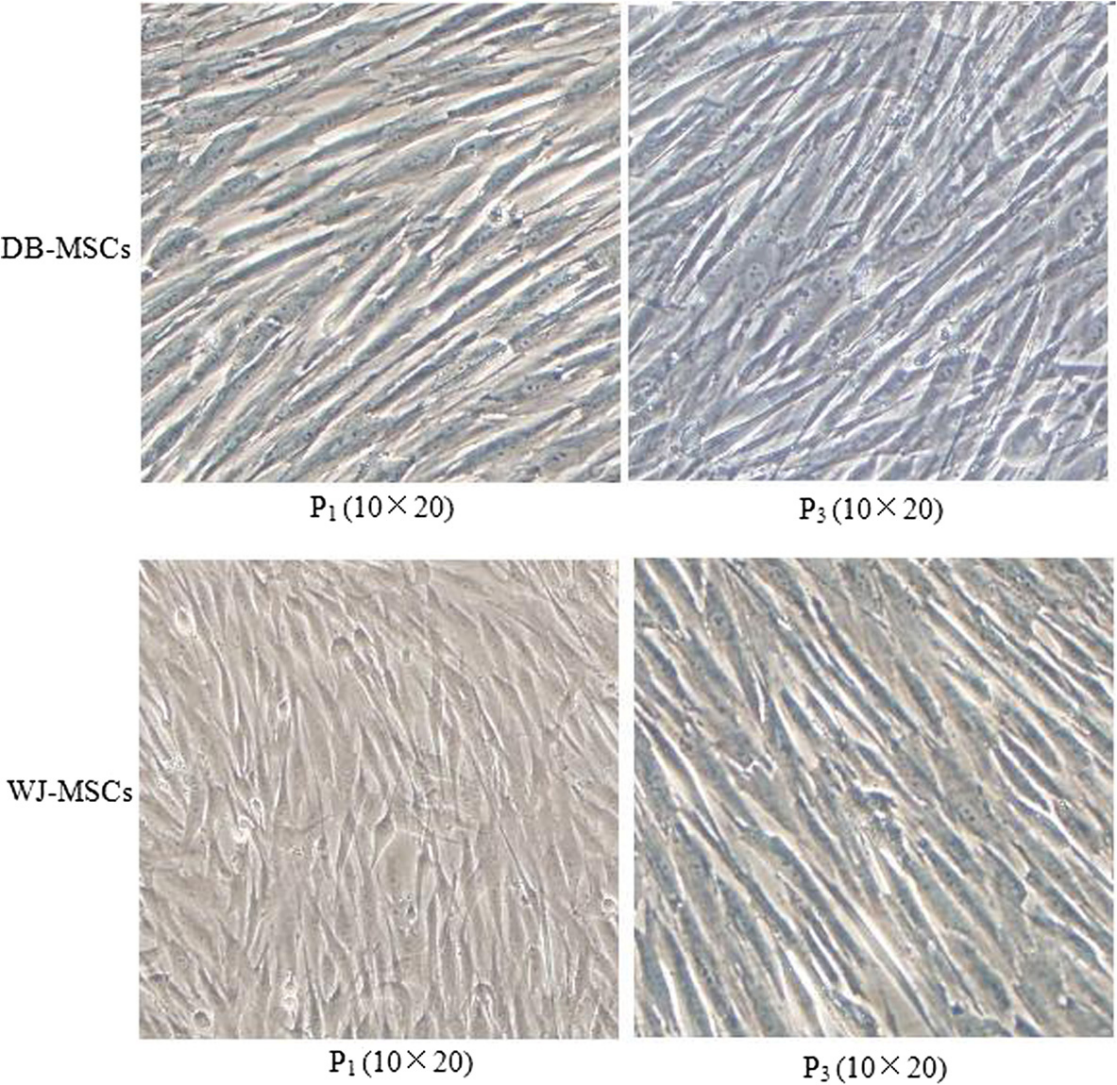
Photomicrographs of mesenchymal stem cells from Wharton’s jelly (*WJ-MSCs*) and the decidua basalis (*DB-MSCs*) from donor 2 are shown. They are plastic-adherent and retain a fibroblast-like morphology. *P* Passage

The morphology of MSCs from both sources was assessed using light microscopy. We observed the cells at every passage. All cells retained a fibroblast-like morphology (Fig. 1).

### Karyotype analysis

**Fig. 2:**
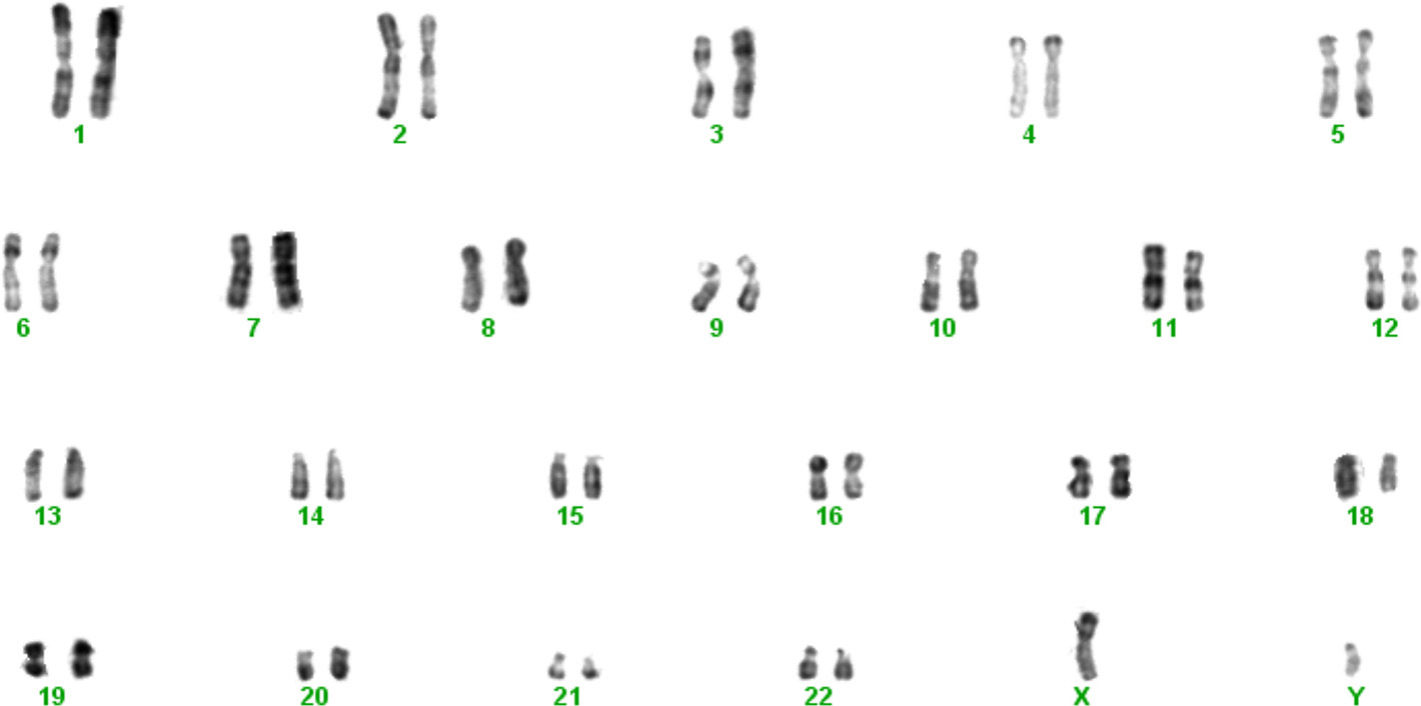
Karyotyping. To ensure all cells in culture were derived from the maternal placenta, the cytogenetic karyotypes of cells at P_0_ were analyzed. The sex chromosomes were XX, not XY. There were no chromosome eliminations, displacements, or imbalances

To ensure all cells in culture were derived from the maternal placenta, the cytogenetic karyotypes of the cells at P_0_ were analyzed. The sex chromosomes XX, not XY, were detected in the cells (Fig. 2).

### Immunophenotype

**Fig. 3:**
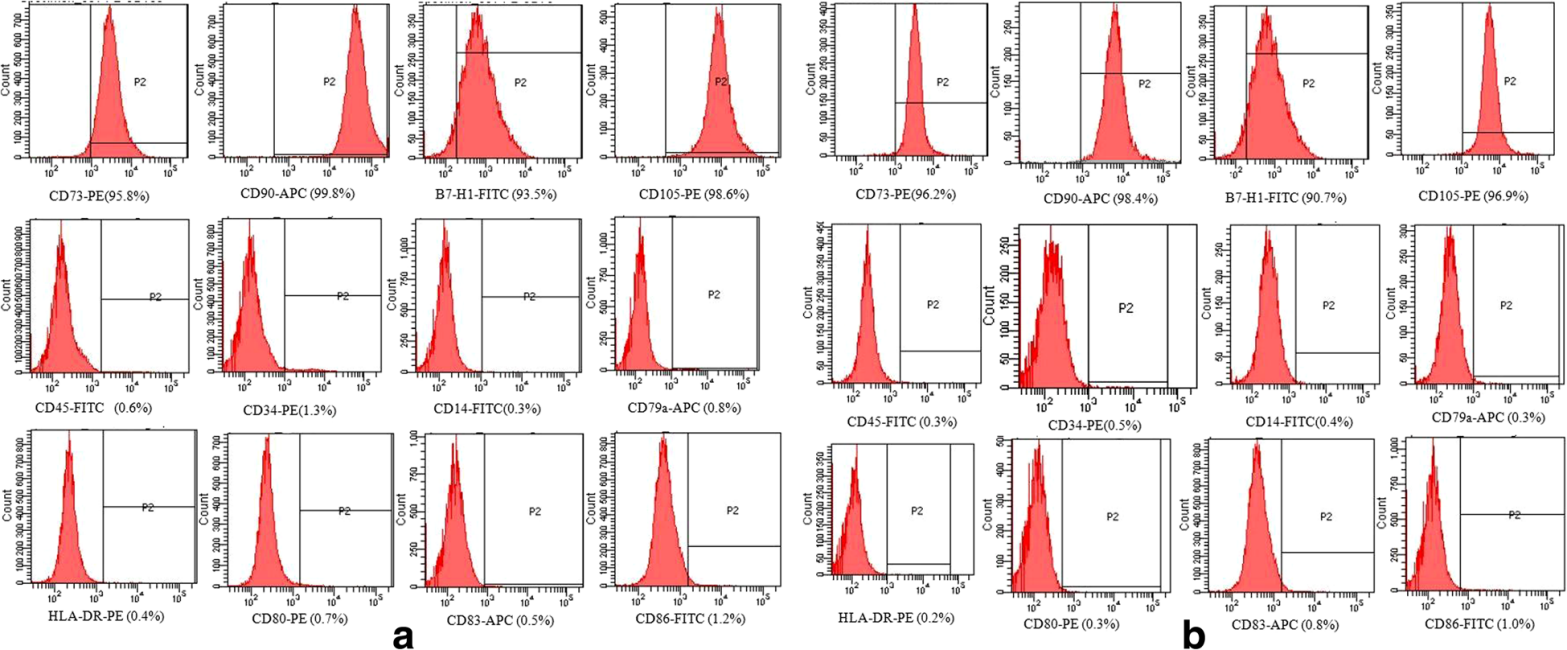
Flow cytometric analysis of the expression of surface markers on (**a**) WJ-MSCs and (**b**) DB-MSCs. The immunofluorescence analysis was conducted at the 3rd passage and showed the immunofluorescence of cells obtained from donor 3. There was no difference between the two types of MSCs in terms of immunophenotype (*n* = 10). *APC* Allophycocyanin, *FITC* Fluorescein isothiocyanate, *PE* Phycoerythrin

We investigated MSC immunophenotype at P_3_ by staining for cell surface markers, which were detected using flow cytometry according to the International Society for Cellular Therapy standards [15]. MSCs from both sources highly expressed the typical MSC markers CD105, CD73, and CD90 and the co-inhibitory molecule B7-H1. In addition, the cells showed low expression of the hematopoietic markers CD45, CD14, and CD34, the MHC class II molecule HLA-DR, and the positive co-stimulatory factors CD80, CD83, and CD86. There was no difference between the two types of MSC in terms of immunophenotype (Fig. 3).

### PDT of MSCs

**Fig. 4:**
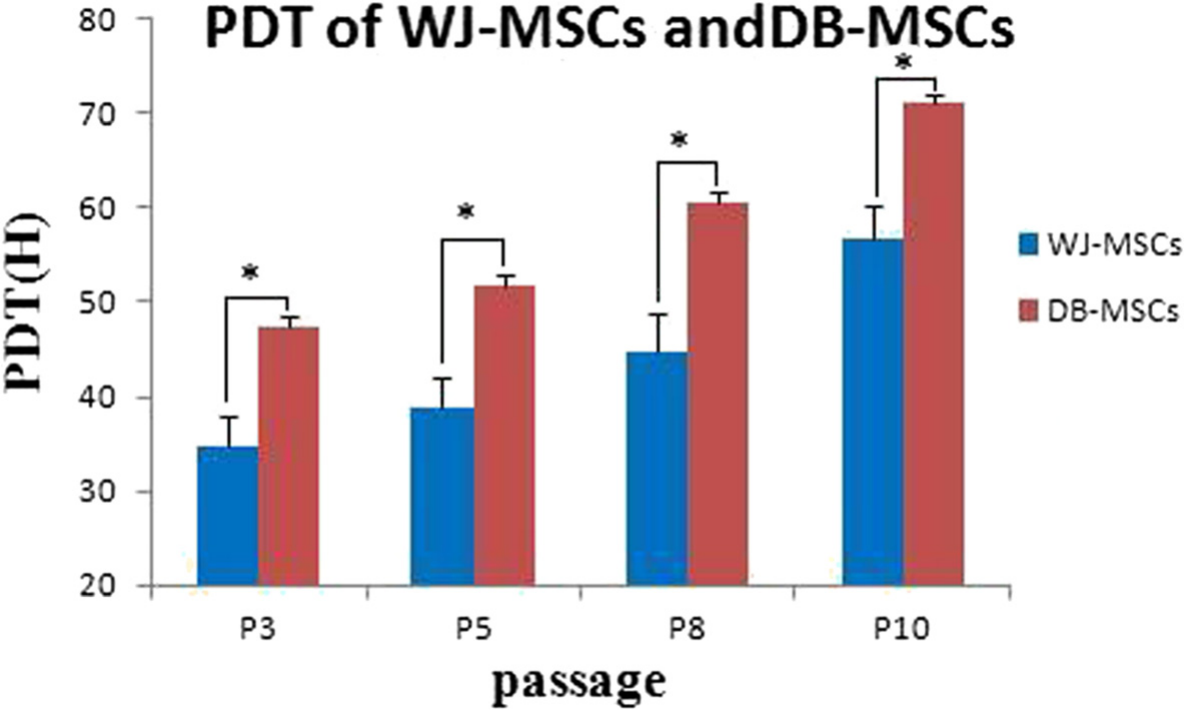
Analysis of the mean population doubling time (*PDT*) of mesenchymal stem cells from Wharton’s jelly (*WJ-MSCs*) and the decidua basalis (*DB-MSCs*) showed that the two types of cells had different proliferative capacities at the same culture passage (*P < 0.05; *n* = 10). The PDT of DB-MSCs and WJ-MSCs obtained from the same donor increased with an increase in the number of passages

DB-MSCs and WJ-MSCs from the same donor showed different proliferative capacities at the same culture passage. The PDT of WJ-MSCs was 34.7 ± 3.4 h, 38.8 ± 3.3 h, 44.8 ± 4.1 h, and 56.8 ± 3.6 h at P_3_, P_5_, P_8_, and P_10_, respectively. The PDT of DB-MSCs was 47.5 ± 4.0 h, 51.8 ± 3.8 h, 60.7 ± 4.7 h, and 71.1 ± 3.0 h at P_3_, P_5_, P_8_, and P_10_, respectively. The PDT of DB-MSCs and WJ-MSCs from the same donor increased with an increase in the number of passages (Fig. 4).

### Cell cycle analysis

**Fig. 5:**
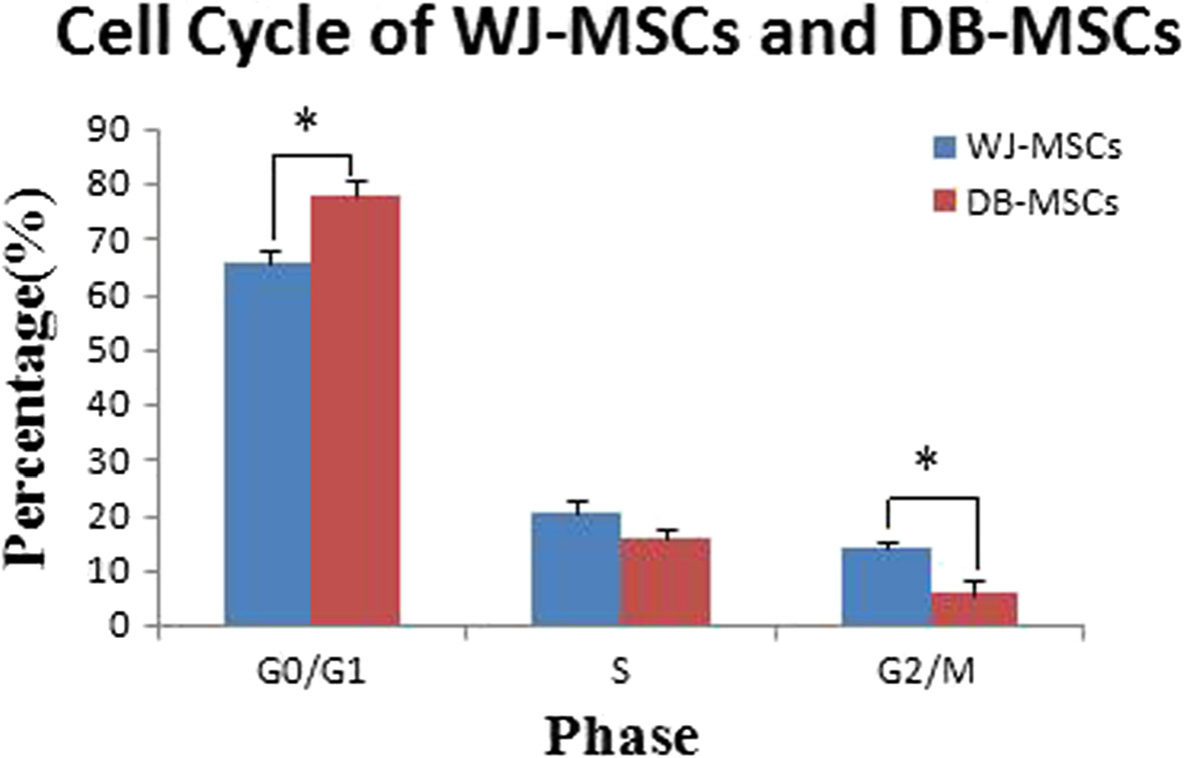
The cell cycles of mesenchymal stem cells from Wharton’s jelly (*WJ-MSCs*) and the decidua basalis (*DB-MSCs*) obtained from the 10 donors were assessed at P_3_. The differences in G0/G1 and G2/M phase distribution between DB-MSCs and WJ-MSCs were statistically significant (*P < 0.05)

The cell cycles of DB-MSCs and WJ-MSCs from the 10 donors were assessed at P_3_. In the case of the DB-MSCs, the mean proportions of cells in the G0/G1 phase, S phase, and G2/M phase were 76.60 ± 2.34 %, 15.76 ± 2.11 %, and 7.64 ± 1.48 %, respectively. The corresponding proportions in the case of WJ-MSCs were 65.615 ± 2.91 %, 20.50 ± 1.96 %, and 13.89 ± 2.78 %. The differences in the distribution of cells in the G0/G1 and G2/M phases between DB-MSCs and WJ-MSCs were statistically significant (*P* < 0.05; Fig. 5).

### Immunomodulatory properties of MSCs from both sources

**Fig. 6:**
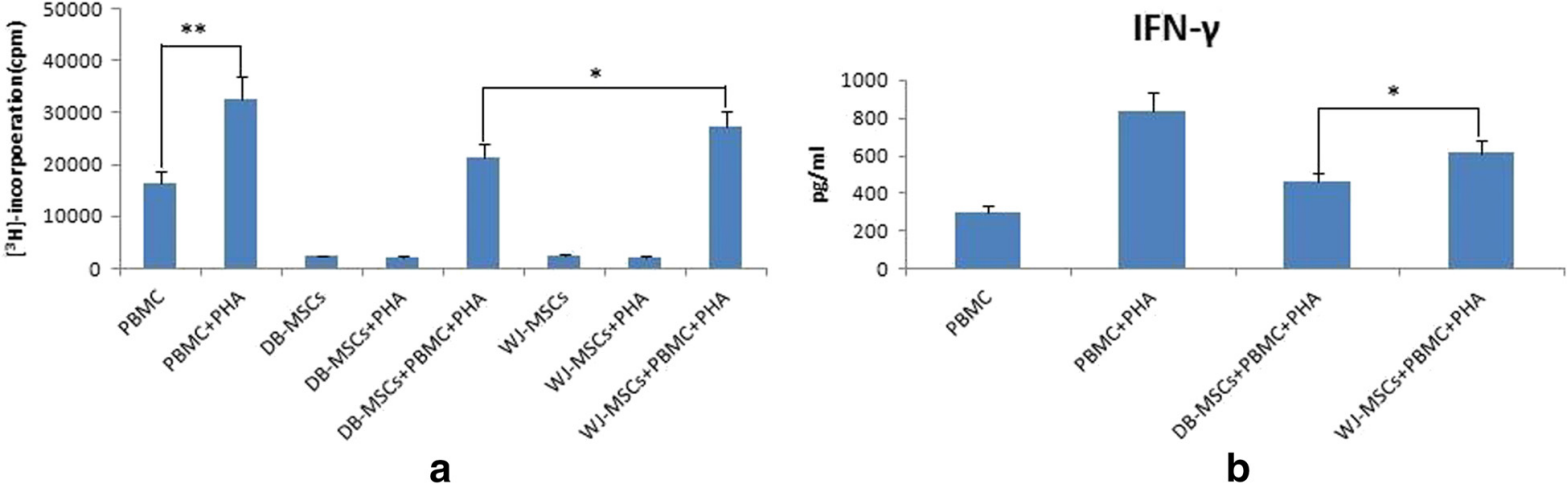
Mesenchymal stem cells from the decidua basalis (*DB-MSCs*) show strong immunosuppressive capacity. **a** The significant difference between groups A and B indicated that phytohemagglutinin (*PHA*) stimulated peripheral blood mononuclear cell (*PBMC*) proliferation (**P < 0.01, *n* = 10). There was no difference between groups C and D or between groups F and G (*P* > 0.05), which indicated that PHA had little effect on the proliferation of MSCs. DB-MSCs showed stronger immunosuppression properties than did mesenchymal stem cells from Wharton's jelly (*WJ-MSCs*) (*P < 0.05). **b** The IFN-γlevel in the supernatantwas lower in the DB-MSC group than in the WJ-MSC group (*P < 0.05)

To compare the immunomodulatory properties of MSCs from both sources, PBMCs were stimulated with PHA in the presence of WJ-MSCs or DB-MSCs for 6 days. Allogeneic PBMC proliferation rates were then assessed using (^3^H)-thymidine incorporation in the four groups. DB-MSCs showed stronger immunosuppression properties than did WJ-MSCs (*P* < 0.05). IFN-γ content of the supernatant was tested using ELISA. The IFN-γ level in the supernatant was lower in the DB-MSC group than in the WJ-MSC group (*P* < 0.05; Fig. 6).

## Discussion

In the present study, we compared two populations of MSCs derived from the decidua basalis and Wharton’s jelly. Although DB-MSCs and WJ-MSCs share global properties, such as morphology, plastic adherence, and multi-lineage differentiation potential [16], significant differences exist between them in terms of growth rate and immunomodulatory function.

During pregnancy, the maternal and fetal immune cells come into direct contact with each other in the decidua, which functions as an immunological barrier between the mother and the developing fetus [5]. Karyotype analysis showed that DB-MSCs are of maternal origin, since the sex chromosomes in these cells were XX, not XY. Programmed cell death (PD)-L1 and PD-L2 are members of the B7 family, and are the ligands for the PD-1 receptor. PD-L1, also called B7-H1, is expressed on antigen-presenting cells, including IFN-γ-stimulated monocytes, and activated human and murine dendritic cells. PD-L1 is also expressed on placental trophoblasts, myocardial endothelium, cortical thymic epithelial cells, and on most carcinomas. Studies show overlapping functions of PD-L1 and PD-L2, and indicate an important role for the PD-L–PD-1 pathway in regulating T-cell responses [17]. The co-inhibitory molecule B7-H1 was highly expressed in DB-MSCs and WJ-MSCs. This molecule may be related to the regulatory function of the cells [18]. Neither cell type expressed the surface MHC class II molecule HLA-DR or positive co-stimulatory molecules, such as CD83, CD80, and CD86. This is consistent with the results of previously published papers [19, 20].

DB-MSCs and WJ-MSCs from the 10 donors exhibited different proliferation rates, and the PDT greatly varied among cells obtained from different donors at the same passage. Shaer et al. [21] compared MSCs from the placental decidua basalis, umbilical cord Wharton’s jelly, and amniotic membrane. The doubling times for WJ-MSCs were 21 ± 8 h at P_3_ and 30 ± 5 h at P_10_, which are shorter than the times determined in this study. This difference may have been caused by the use of different culture systems, i.e., serum-free versus serum-containing cultures. The authors of the above study also reported that the proliferative potential of WJ-MSCs tended to be higher than that of the cells from the other two sources. Overall, WJ-MSCs exhibited higher growth rates than did DB-MSCs under the same conditions. The results of cell cycle assessments agreed with those of the PDT analysis.

The fetal–maternal interface seems to be immunologically special to enable maternal acceptance of the fetal allograft [22]. The human placenta, besides supporting fetal development, may also function as an immune regulator. MSCs are anti-proliferative to T cells and suppress the secretion of IFN-γ in mixed lymphocyte reaction cultures [23]. Karlsson et al. [24] compared stromal cells obtained from term fetal membrane, umbilical cords, and placental villi, and found that the stromal cells obtained from term fetal membrane had stronger immunosuppressive capacity than those from umbilical cords and placental villi. DB-MSCs produced significantly lower levels of IFN-γ than did WJ-MSCs. The mechanisms of T-cell immunosuppression by MSCs has always been an issue of dispute. Toll-like receptors are considered to play a key role in this process [25–27]. MSCs immunoregulate T-cell proliferation independent of heme oxygenase-1 [28].

## Conclusion

In this study, we compared the essential biological characteristics of DB-MSCs and WJ-MSCs. Although the two cell types share global properties, such as morphology, plastic adherence, and multi-lineage differentiation potential, WJ-MSCs exhibited higher growth rates, and DB-MSCs had stronger immunomodulatory function. Better treatment effects may be obtained if the characteristics of MSCs from different sources and the aim of the clinical application are considered.

## Abbreviations

APC: Allophycocyanin
DB-MSC: Mesenchymal stem cell from the decidua basalis
ELISA: Enzyme-linked immunosorbent assay
FITC: Fluorescein isothiocyanate
IFN: Interferon
MHC: Major histocompatibility complex
MSC: Mesenchymal stem cell
P: Passage
PBMC: Peripheral blood mononuclear cell
PD: Programmed cell death
PDT: Population doubling time
PE: Phycoerythrin
PHA: Phytohemagglutinin
WJ-MSC: Mesenchymal stem cell from Wharton’s jelly
